# Genome Sequence of the Acetogenic Bacterium *Acetobacterium wieringae* DSM 1911^T^

**DOI:** 10.1128/genomeA.01430-16

**Published:** 2016-12-22

**Authors:** Anja Poehlein, Frank Robert Bengelsdorf, Bettina Schiel-Bengelsdorf, Rolf Daniel, Peter Dürre

**Affiliations:** a Genomic and Applied Microbiology and Göttingen Genomics Laboratory, Georg-August University Göttingen, Göttingen, Germany; b Institut für Mikrobiologie und Biotechnologie, Universität Ulm, Ulm, Germany

## Abstract

Here, we report the draft genome sequence of *Acetobacterium wieringae* DSM 1911^T^, an anaerobic, autotrophic, acetogenic, d,l-lactate-utilizing bacterium. The genome consists of a chromosome (3.88 Mb) and 3,620 predicted protein-encoding genes.

## GENOME ANNOUNCEMENT

*Acetobacterium wieringae* DSM 1911^T^ is a Gram-positive, anaerobic, and acetogenic bacterium, which was isolated from mud of the Göttingen, Germany, sewage plant and described by Braun and Gottschalk in 1982 (1). The strain was shown to produce acetate, formate, and ethanol during autotrophic batch cultivation in a continuously stirred-tank bioreactor (2). The genus *Acetobacterium* includes several autotrophic acetogenic bacterial species such as *A. bakii*, *A. carbinolicum*, *A. fimetatium*, *A. malicum*, *A. paludosum*, and *A. woodii* (3). In addition to their ability to grow autographically (consuming CO_2_ and H_2_) using the Wood-Ljungdahl pathway, all strains can also utilize lactate for growth. Weghoff et al. (4) showed that the lactate dehydrogenase (LDH) of *A. woodii* forms a stable complex with an electron-transferring flavoprotein (Etf) that can reduce NAD^+^ in the presence of reduced ferredoxin (Fd^2−^).

Chromosomal DNA of *A. wieringae* was isolated using the MasterPure Gram-positive DNA purification kit (Epicentre, Madison, WI, USA). Illumina paired-end sequencing libraries were generated from the extracted DNA according to the protocol of the manufacturer (Illumina, San Diego, CA, USA). Sequencing was performed using a MiSeq instrument and the MiSeq reagent kit version 3, as recommended by the manufacturer (Illumina), resulting in 2,191,792 paired-end reads (301 bp). Trimmomatic version 0.32 (5) was used for quality filtering. This resulted in 2,036,834 remaining high-quality paired-end reads. The *de novo* assembly was performed with the SPAdes genome assembler software version 3.5.0 (6). The assembly resulted in 62 contigs (>500 bp) with an average coverage of 106-fold. QualiMap version 2.1 (7) was used to validate the assembly and to determine the read coverage. The draft genome of *A. wieringae* comprises a circular chromosome (3,895,828 bp) with an overall G+C content of 44.07%. Automatic gene prediction and identification of rRNA and tRNA genes were performed using the software tool Prokka (8). The genome contains six rRNA genes, 39 tRNA genes, 2,704 protein-encoding genes with predicted functions, and 916 genes coding for hypothetical proteins.

Analysis of the genome and comparison with the *A. woodii* genome sequence (9) revealed that *A. wieringae* harbors the identical gene cluster responsible for lactate utilization (*lctABCDEF*) as *A. woodii* (4, 9). The genome of* A. wieringae* also contains two complete gene clusters for the *Rnf* (*Rhodobacter* nitrogen fixation) complex, whereas the genomes of almost all other autotrophic acetogenes harbor only one *Rnf* cluster. To date, only the *Clostridium magnum* genome is known to encode two complete gene clusters for the *Rnf* complex (10). *A. wieringae* contains a gene cluster encoding proteins for the carbonyl branch of the Wood-Ljungdahl pathway which is identical to that of *A. woodii*. In addition, the gene clusters encoding proteins for the methyl branch of the Wood-Ljungdahl pathway are similar in the genomes of both organisms. However, *A. wieringae* lacks genes encoding the glycine cleavage H protein and dihydrolipoamide dehydrogenase (11).

### Accession number(s).

This whole-genome shotgun project has been deposited at DDBJ/EMBL/GenBank under the accession number LKEU00000000. The version described in this paper is the first version, LKEU01000000.

## References

[1] Braun M, Gottschalk G 1982 *Acetobacterium wieringae* sp. nov., a new species producing acetic acid from molecular hydrogen and carbon dioxide. Zbl Bakt Hyg, I Abt Orig C3:368–376.

[2] Groher A, Weuster-Botz D 2016 Comparative reaction engineering analysis of different acetogenic bacteria for gas fermentation. J Biotechnol 228:82–94. doi:10.1016/j.jbiotec.2016.04.032. 27107467

[3] Bengelsdorf FR, Straub M, Dürre P 2013 Bacterial synthesis gas (syngas) fermentation. Environ Technol 34:1639–1651. doi:10.1080/09593330.2013.827747. 24350425

[4] Weghoff MC, Bertsch J, Müller V 2015 A novel lactate metabolism in strictly anaerobic bacteria. Environ Microbiol 17:670–677. doi:10.1111/1462-2920.12493. 24762045

[5] Bolger AM, Lohse M, Usadel B 2014 Trimmomatic: a flexible trimmer for Illumina sequence data. Bioinformatics 30:2114–2120. doi:10.1093/bioinformatics/btu170. 24695404 PMC4103590

[6] Bankevich A, Nurk S, Antipov D, Gurevich AA, Dvorkin M, Kulikov AS, Lesin VM, Nikolenko SI, Pham S, Prjibelski AD, Pyshkin AV, Sirotkin AV, Vyahhi N, Tesler G, Alekseyev MA, Pevzner PA 2012 SPAdes: a new genome assembly algorithm and its applications to single cell sequencing. J Comput Biol 19:455–477. doi:10.1089/cmb.2012.0021. 22506599 PMC3342519

[7] García-Alcalde F, Okonechnikov K, Carbonell J, Cruz LM, Götz S, Tarazona S, Dopazo J, Meyer TF, Conesa A 2012 Qualimap: evaluating next-generation sequencing alignment data. Bioinformatics 28:2678–2679. doi:10.1093/bioinformatics/bts503. 22914218

[8] Seemann T 2014 Prokka: rapid prokaryotic genome annotation. Bioinformatics 30:2068–2069. doi:10.1093/bioinformatics/btu153. 24642063

[9] Poehlein A, Schmidt S, Kaster AK, Goenrich M, Vollmers J, Thürmer A, Bertsch J, Schuchmann K, Voigt B, Hecker M, Daniel R, Thauer RK, Gottschalk G, Müller V 2012 An ancient pathway combining carbon dioxide fixation with the generation and utilization of a sodium ion gradient for ATP synthesis. PLoS One 7:e33439. doi:10.1371/journal.pone.0033439. 22479398 PMC3315566

[10] Uhlig R, Poehlein A, Fischer RJ, Daniel R, Bahl H 2016 Genome sequence of the autotrophic acetogen *Clostridium magnum* DSM 2767. Genome Announc 4(3):e00464-16. doi:10.1128/genomeA.00464-16. 27284147 PMC4901216

[11] Poehlein A, Cebulla M, Ilg MM, Bengelsdorf FR, Schiel-Bengelsdorf B, Whited G, Andreesen JR, Gottschalk G, Daniel R, Dürre P 2015 The complete genome sequence of *Clostridium* *aceticum*: a missing link between Rnf- and cytochrome-containing autotrophic acetogens. mBio 6:e01168–e01115. doi:10.1128/mBio.01168-15. 26350967 PMC4600107

